# F149. NEUROBIOLOGY OF SELF-AGENCY DURING REALITY MONITORING AND SPEECH FEEDBACK MONITORING: IMPLICATIONS FOR TREATMENT DEVELOPMENT IN SCHIZOPHRENIA

**DOI:** 10.1093/schbul/sby017.680

**Published:** 2018-04-01

**Authors:** Karuna Subramaniam, Leighton Hinkley, Hardik Kothare, Danielle Mizuiri, Sophia Vinogradov, John Houde, Srikantan Nagarajan

**Affiliations:** 1 Univeristy of California, San Francisco; 2 University of Minnesota

## Abstract

**Background:**

Self-agency is the experience of being the agent of one’s own thoughts and motor actions. The intact experience of self-agency is necessary for successful interactions with the outside world (i.e., reality monitoring). Reality monitoring is the ability to distinguish internally self-generated information from outside reality (externally-derived information). We found that healthy control (HC) participants recruit medial prefrontal cortex (mPFC) during encoding of self-generated information, which is also activated during accurate retrieval of self-generated information. By contrast, patients with schizophrenia (SZ) have specific self-agency impairments and do not show mPFC activation during encoding or retrieval of self-generated information. These findings indicate that SZ may rely more on environmental externally-derived information, rather than on internal self-generated information, to guide reality monitoring. Here, we relate the experience of self-agency during a lower-level speech feedback monitoring (i.e., monitoring what we hear ourselves say) to our higher-level cognitive reality monitoring task. We examine whether the sense of self-agency during speech feedback monitoring and reality monitoring are driven by the same fundamental mechanism that we hypothesize underlies the capacity to experience self-agency—the ability to make reliable predictions about the outcomes of self-generated actions.

**Methods:**

During speech feedback monitoring we assess self-agency by altering environmental auditory feedback so that participants listen to a perturbed version of their own speech. When subjects hear minimal perturbations in their auditory feedback while speaking, they make corrective responses, indicating that they judge the perturbations as errors in their speech output. These corrective responses are modulated by subjects’ reliance on internal predictions about the outcome of their speech output; the more subjects rely on their internal predictions, i.e. their sense of self-agency, the less they rely on external auditory feedback, resulting in smaller corrective responses. Thus, subjects who produced smaller corrective responses manifested an enhanced sense of self-agency in that they relied more on their internal predictions to generate their own actions (i.e., their speech output).

**Results:**

We found that self-agency judgments in the reality-monitoring task were higher in people who had smaller corrective responses (p=.05) during minimal speech perturbations of their auditory feedback. These results provide support for a unitary process for the experience of self-agency resulting from the ability to reliably predict the outcomes of self-generated actions, that governs low-level speech control and higher level reality monitoring.

**Discussion:**

These findings have important therapeutic implications in SZ, suggesting that the more participants rely on internal predictions to guide their actions, the smaller their corrective responses in their speech output, and the more likely they are to make correct judgments of self-agency during reality monitoring. In conclusion, these findings, therefore, indicate that reality monitoring and speech monitoring paradigms provide quick and robust markers of the experience of self-agency, indicating which subjects followed their internal predictions to guide their own actions. Together, these findings have important therapeutic implications for potentiating improvements in self-agency judgments not only in HC, but in patients with schizophrenia who suffer from critical self-agency impairments.

